# BARRIERS TO HOSPICE CARE TRANSITIONS AMONG PEOPLE WITH DEMENTIA IN HOME HEALTH CARE: VIEWS OF DIVERSE CARE PARTNERS

**DOI:** 10.1093/geroni/igae098.2058

**Published:** 2024-12-31

**Authors:** Komal Murali, Gwenneth Wang, Sasha Vergez, Margaret McDonald, Dena Schulman-Green, Abraham Brody, Karen Bullock

**Affiliations:** New York University, New York, New York, United States; New York University, New York, New York, United States; VNS Health, New York, New York, United States; Visiting Nurse Service of New York, New York, New York, United States; New York University, New York, New York, United States; HIGN, New York University Rory Meyers College of Nursing, New York, New York, United States; Boston College, Chestnut Hill, Massachusetts, United States

## Abstract

Racially and ethnically minoritized persons living with dementia (PLWD) use hospice at disparately low rates and home healthcare (HHC), a preferred setting of end-of-life care, is a key intervention point for increasing hospice transitions. We conducted qualitative interviews with diverse care partners of PLWD to explore views about barriers to hospice care and experiences with dementia care and end-of-life decision-making in HHC. Conceptually rooted in the NIMHD Health Disparities and Equity-Focus Implementation Research Frameworks, we conducted directed content analysis. Participants (n=20) were mostly female adult children of PLWD identifying as Black, Hispanic/Latino, Asian, or White. Six main concepts emerged: 1) Despite general openness to hospice, there was variable knowledge including misconceptions that it could not be received in the home; 2) Limited knowledge of dementia illness trajectories and end-of-life care options including hospice; 3) Communication challenges and conflicts associated with family-decision making; 4) Difficulty with care coordination and limitations of health insurance coverage and access to dementia caregiving support and resources; 5) Unmet cultural aspects of care and language barriers (language discordance between aides and PLWD) led to conflict between care partners and HHC staff); 6) Desire to honor and balance prior stated wishes of PLWD (e.g., prioritizing care at home, avoiding hospitalizations and nursing home) with end-of-life dementia care and decision-making. Findings shed light on views related to hospice care barriers and decision-making experiences of diverse care partners of PLWD that are key for the development of equity-focused culturally sensitive end-of-life care in HHC.

